# Clinical results after microincision biaxial cataract surgery and implantation of an Incise intraocular lens

**DOI:** 10.1007/s10792-017-0686-0

**Published:** 2017-08-12

**Authors:** Wojciech Lubiński, Marta Kirkiewicz, Karolina Podborączyńska-Jodko

**Affiliations:** Clinic of Ophthalmology, Pomeranian Medical University, al. Powstańców Wielkopolskich 72, 70-111 Szczecin, Poland

**Keywords:** Biaxial microincision cataract surgery, Microincision intraocular lens, Surgically induced astigmatism, Spherical equivalent refraction, Contrast sensitivity

## Abstract

**Purpose:**

To evaluate clinical outcomes after uncomplicated microincision biaxial cataract surgery and implantation of Incise intraocular lens (IOL).

**Methods:**

This study included 47 eyes of 29 patients (mean age 62.2 ± 8.6 years), who underwent 1.4-mm biaxial cataract surgery with implantation of the Incise IOL (Bausch and Lomb). At third month, surgically induced astigmatism (SIA) was calculated. Three, 6 and 12 months postoperatively, uncorrected distance visual acuity (UDVA), corrected distance visual acuity (CDVA), corrected near visual acuity (CNVA) LogMAR ETDRS, spherical equivalent refraction (SER), photopic distance corrected contrast sensitivity (CS) with and without glare (85 cd/m^2^) (CSV-1000) were assessed. One year after surgery, late complications were assessed and subjects were questioned for subjective symptoms.

**Results:**

Mean of SIA was equal 0.29 ± 0.16 D. Three months postoperatively: mean UDVA improved from 0.83 to 0.04 (*p* < 0.001), CDVA from 0.58 to −0.05 (*p* < 0.001) and CNVA from 0.58 to −0.02 (*p* < 0.001) and all were stable during 1-year follow-up. Three months postoperatively, the mean SER was equal 0.07 ± 0.61 D and was within ±0.5 D in 79%, and within 1 D in 88% of eyes. During follow-up period, corrected CS with and without glare for distance was found to be within normal limits. The only late complication was posterior capsule opacification (PCO). Subjective quality of vision was very high; none of patients complained about glare.

**Conclusions:**

Biaxial cataract surgery with implantation of the Incise IOL provided excellent clinical outcomes by minimizing SIA, stable refraction and low incidence of PCO.

## Introduction

Microinvasive surgical methods determine the development direction of today’s ophthalmology. Examples of these trends are MIVS (microincision vitrectomy surgery), MIGS (microinvasive glaucoma surgery) and MICS (microincision cataract surgery).

The goal of MICS is to treat cataract using 2.0 or smaller corneal incision with the purpose of reducing surgical invasiveness, improving at the same time surgical outcomes [1]. MICS is divided into two methods: coaxial MICS (C-MICS) and biaxial MICS (B-MICS). C-MICS gives the possibility to implant the IOL through 1.8 mm incision. The method which reduces this size is B-MICS, which allows 1.4 mm cataract surgery and IOL implantation. B-MICS resulted from the interplay of several factors including microcorneal incisions, bimanuality, improved use of fluids, rapidly progressing instrumentation, and adequate use of low-energy ultrasound phacoemulsificators [2], including development of new sleeveless tip [3, 4]. Advantages for this procedure are lower surgically induced astigmatism (SIA), less postoperative higher-order corneal aberrations (HOA) and lower endothelial cell loss [5–7]. Furthermore, it seems reasonable to conclude that smaller incision size has lower impact on corneal biomechanics, shortens recovery time and reduces number of endophthalmitis, although until now there is no clear evidence to support these hypotheses.

The Incise monofocal intraocular lens (IOL, Bausch & Lomb) is the first innovative IOL that can be implanted without a convention tunnel preparation but through 1.4 mm paracentesis. In the available literature, only three promising study results with short follow-up period up to 6 months were published on clinical outcomes of this lens [5–7]. Above-mentioned studies were performed with biaxial technique with IOL implantation from 1.4 mm [5] and 1.6 mm [6] clear cornea incision and one with coaxial microincision (C-MICS), where the lens was implanted through 1.8 mm incision [7].

This is why we decided to assess clinical outcomes after B-MICS with implantation of these lenses in longer follow-up period of 12 months.

## Methods

### Patients

This consecutive prospective observational noncomparative clinical trial included 47 eyes of 29 patients, 18 women and 11 men. Informed consent was obtained prior to treatment from each patient. The patients’ mean age was equal 62.2 ± 8.6 (range 43–76 years). The cataract grade (LOCS III) ranged from No. 3–6 [8]. None of the eyes had associated ocular diseases that might affect the visual outcome postoperatively. The study adhered to the tenets of the Declaration of Helsinki and was approved by the local ethics committee. Informed consent was obtained from all individual participants included in the study.

Biometry was performed on IOLMaster (Carl Zeiss). Ultrasound biometry was utilized in eyes in which IOLMaster could not be performed because of a dense cataract. The IOL power was calculated with SRK-T formula (A-constant 118.9) in eyes with the axial length (AL) of 22–24 mm. Hoffer Q formula (pACD = 5.61) was used in eyes with a shorter AL (<22 mm).

### Examination protocol

All patients had preoperatively full complete ophthalmologic examination including monocular uncorrected distance visual acuity (UDVA), corrected distance visual acuity (CDVA) (4 m), corrected near visual acuity (CNVA) (LogMAR) (40 cm), measured on Early Treatment Diabetic Study (ETDRS) charts, 3, 6 and 12 months postoperatively following parameters were assessed: UDVA, CDVA and CNVA, spherical equivalent refraction (SER), photopic corrected contrast sensitivity (CS) with and without glare (85 cd/m^2^) (CSV-1000E instrument), measured at the distance of 2 m. At third month, surgically induced astigmatism was calculated using vector analysis based on the keratometry results, which was described by Dr. Saurabh Sawhney and Dr. Aashima Aggarwal [9, 10]. One year after surgery late complications were assessed. Additionally patients were questioned for subjective symptoms (modified National Eye Institute Visual Function Questionnaire-14; NEI VFQ-14).

### Statistical analysis

Distribution of analyzed data was performed using the Kolmogorov–Smirnov test. All data presented in the current study were not normally distributed, and therefore nonparametric statistics were used. Wilcoxon ranked sum test was used to compare changes in visual acuities and astigmatism between preoperative and postoperative examinations, considering a significance level of *p* < 0.05. The statistical analysis was performed using Statistica StatSoft^®^.

### The intraocular lens

The Incise IOL (Bausch & Lomb) is a single piece aspheric hydrophilic, aberration free IOL and has sharp edge radius (less than 5 µm) and a continuous 360 square-edge profile—design features that help to prevent cell migration and decrease the incidence of posterior capsular opacification (PCO). The acrylic lens material has 22% of water content and contains more hydrophobic monomers than other acrylic IOL materials, making Incise resistant to tears and other surgical trauma. The combination of unique material and single-use injector system enables the IOL to be implanted through an incision as small as 1.4 mm.

### Surgical technique

All surgeries were performed by one surgeon (WL). Topical (proxymetacaine hydrochloridum) and local (1% lidocaine) anesthesia was used. Low-power phacoemulsification systems were used to emulsify the cataract through 2 microincisions in the 2 and 10 o’clock positions using trapezoidal knife 1.2 × 1.4 mm. The continuous curvilinear capsulorhexis of 5 mm diameter, hydrodissection and hydrodelineation under protection with Discovisc^®^ were performed. The Stellaris Vision Enhancement System (Bausch & Lomb, Rochester, N.Y., USA) was used for phacoemulsification with mean parameters: burst mode—mean power 4.69 ± 4.39% (SD), mean effective phacotime 0.78 ± 2.22 s (SD). Nucleus was divided into two or four pieces using Phaco PreChopper. The IOL was implanted through the clear cornea incision in superotemporal quadrant with a “wound-assist” technique [2]. The size of incisions after IOL implantation was measured with calipers (Asico).

## Results

### Corneal incision size

The mean corneal incision size used for the IOL implantation during the surgery with IOL implantation was 1.44 ± 0.1 mm (range 1.4–2.0 mm). Final incision diameter was 1.5 or smaller in 45 eyes and 1.7 mm in 1 eye. Through these incisions, all the intraocular maneuvers and IOL implantation were performed without the need for further enlargement (except for one case, described further on). The implantation process was estimated as difficult in one eye after the original incision (1.4 mm) had been made and cataract had been removed. In this case, IOL incarcerated in the wound and after incision enlargement the same IOL was implanted through 2.0 mm incision.

### Surgically induced astigmatism (SIA)

**Fig. 1:**
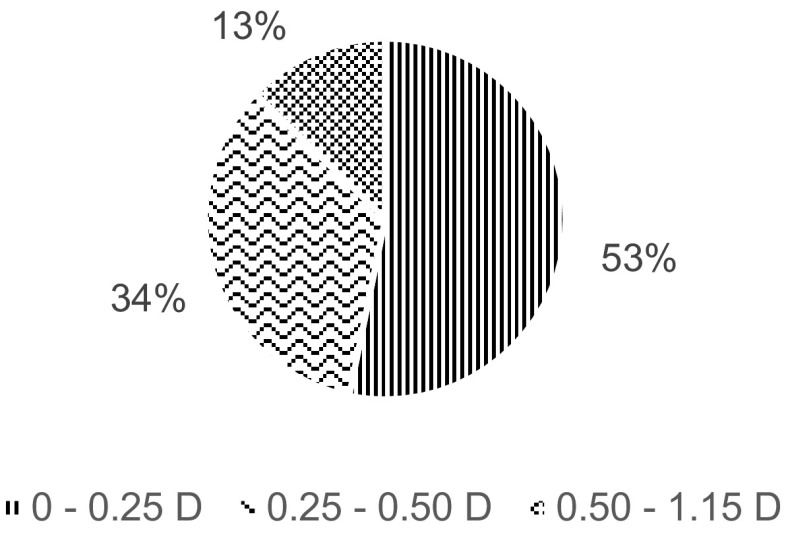
Frequency of surgical induced astigmatism

Mean SIA measured at third month was equal 0.29 ± 0.16 D. Fifty-three percent of eyes had SIA lower than 0.25 D, in 34% of eyes SIA was between 0.25 and 0.5 D, and only in 13% of eyes SIA was higher than 0.5 D (Fig. 1). Before surgery, mean astigmatism was equal 0.67 ± 0.45 D, decreased to 0.64 ± 0.40 D postoperatively and the difference was not statistically significant (*p* > 0.05).

### Distance visual acuity

Comparisons of UDVA and CDVA preoperative and 3, 6, 12 months postoperatively are shown in Table 1.

**Table 1:** Comparisons of uncorrected and best corrected distance visual acuities pre- and postoperatively

Parameters	Mean ± SD				*p* value
	Preoperative	Postoperative			
		3 months	6 months	12 months	
UDVA	0.83 ± 0.59	0.04 ± 0.14	0.10 ± 0.16	0.07 ± 0.15	<0.001
CDVA	0.58 ± 0.57	−0.05 ± 0.06	−0.03 ± 0.05	−0.04 ± 0.05	<0.001

UDVA uncorrected distance visual acuity; CDVA corrected distance visual acuity; SD standard deviation

On the first day after the surgery, mean CDVA was already equal 0.13 ± 0.18. Distance visual acuity was very good in the third month and was stable up to 12-month follow-up examination.

Significant increase of UDVA and CDVA was obtained 3, 6 and 12 months postoperatively in comparison with values before surgery (Table 1).

**Fig. 2:**
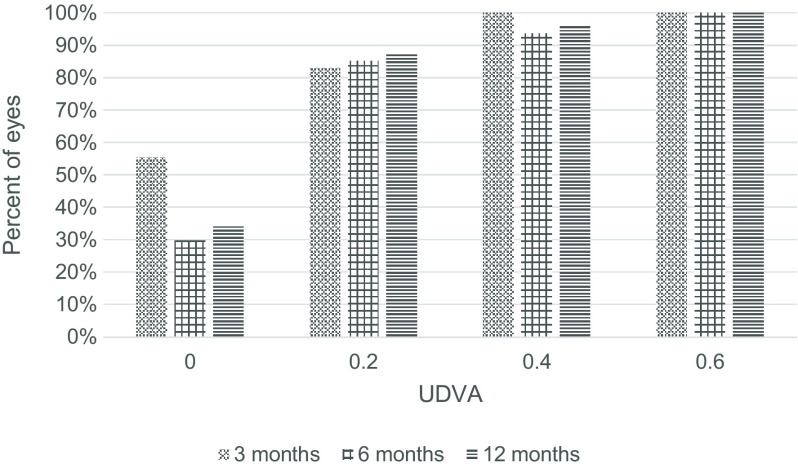
Distribution of the postoperative uncorrected distance visual acuity. *UDVA* Uncorrected distance visual acuity

At third month, UDVA was 0.2 or better in 83% of cases, BDVA was 0.2 or better in 87% of cases (Fig. 2).

### Near visual acuity

Corrected near visual acuity in third, sixth and 1 year postoperatively was significantly better compared to preoperative value (Table 2).

**Table 2:** Comparison of corrected near visual acuity preoperative and postoperative

Parameters	Mean ± SD				*p* value
	Preoperative	Postoperative			
		3 months	6 months	12 months	
CNVA	0.58 ± 0.52	−0.02 ± 0.04	−0.01 ± 0.05	−0.02 ± 0.06	<0.001

CNVA Corrected near visual acuity; SD standard deviation

### Absolute refractive error

**Fig. 3:**
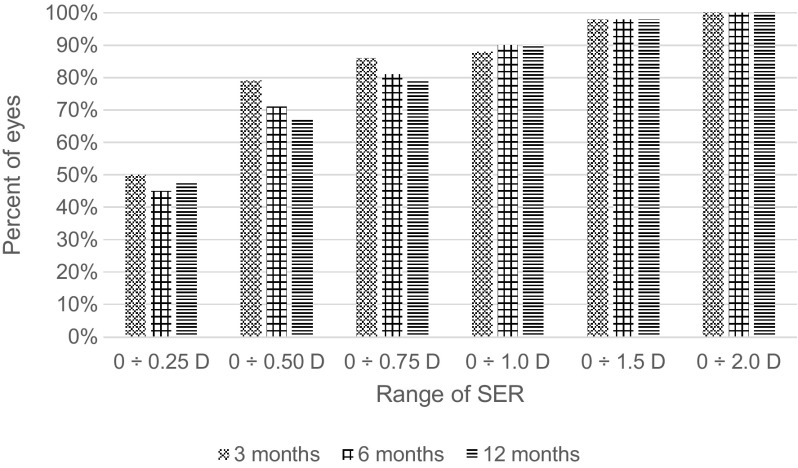
Distribution of postoperative spherical equivalent refraction. *SER* Spherical equivalent refraction

The mean SER was equal 0.07 ± 0.61 D 3 months postoperatively; −0.03 ± 0.64 D 6 months postoperatively and −0.02 ± 0.66 D 12 months after the operation. Regarding predictability, the SER was within ±0.5 D in 79% of eyes and within ±1 D in 88% of eyes at the postoperative third month. Twelve months after surgery in 79% of eyes SER was lower than 0.75 D (Fig. 3).

### Contrast sensitivity

**Fig. 4:**
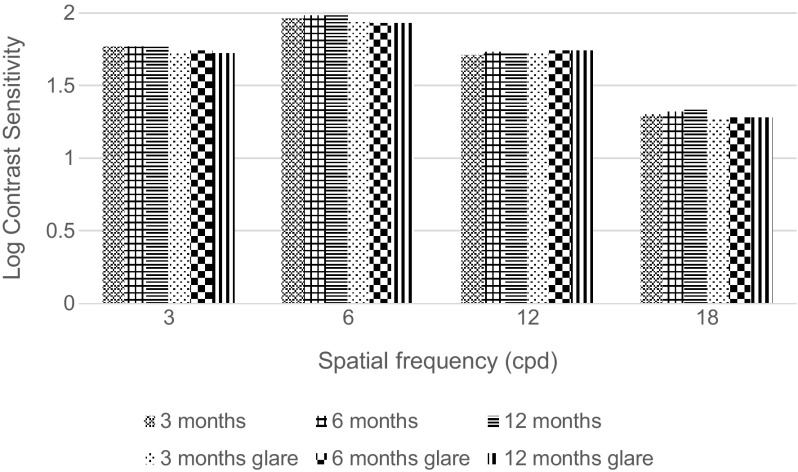
Contrast sensitivities at follow-up visits at each spatial frequency without and with glare. *Cpd* Cycle per degree

During follow-up period, corrected CS with and without glare for distance was found to be within normal limits in comparison with the normal population in the range of 50–75 years [11]. Corrected CSs were almost equal between follow-up visits (Fig. 4).

### Patient’s satisfaction and subjective symptoms

Patient’s satisfaction was very high (Table 3). None of patients complained about glare.

**Table 3:** Patient’s satisfaction—modified Visual Function Questionnaire-14

Questions	Mean (range)
General satisfaction from the procedure	9.6 (0 ÷ 10)
Do you have any difficulty (in glasses) reading traffic, street and a store signs?	100 (0 ÷ 100)
Do you have any difficulty (in glasses) reading newspapers?	100 (0 ÷ 100)
Do you have any difficulty (in glasses) daytime car driving?	100 (0 ÷ 100)
Do you have any difficulty (in glasses) nighttime car driving?	99 (0 ÷ 100)
Do you need glasses for distance?	15%
Do you need glasses for near?	98%
Do you have night vision problems?	2%

A score of 100 indicates able to do all applicable activities; a score of 0 indicates unable to do all applicable activities because of vision disturbances

### Complications

The incident of corneal wound leakage was noticed in one case at first day after the cataract surgery. It was treated with contact lens and in day 4 the wound was stable without need for additional treatment. During 1-year follow-up, no incident of PCO which was a cost of visual acuity was noticed and there was no need for Nd: Yag procedure.

## Discussion

Microincision cataract surgery develops rapidly. New techniques like B-MICS and C-MICS procedures significantly lowered mean phacoemulsification time, mean phacoemulsification power and surgically induced astigmatism when compared with standard coaxial phacoemulsification [12]. In B-MICS technique, irrigation and aspiration are performed separately. Through two opposite 1.2–1.4 mm paracentesis, the phacotip without sleeve and the irrigation tip are inserted into the anterior chamber. C-MICS technique requires tunnel and two additional paracentesis and the only difference from traditional coaxial phaco is the use of 21-gauge phacotip. During B-MICS procedure, the tip without sleeve is used and it is possible to damage the cornea around the wound because of possible thermal burn [13]. It may cause “leaky wound” and in consequences opens the way to bacterial infiltration from conjunctival sac. In the present work, one incident of corneal leakage at first day after the cataract surgery was noticed. It was treated with contact lens, and in day 4 the wound was stable without need for additional treatment. Other authors report anterior chamber instability during the surgery [5]. In our series of patients, where the Stellaris Phaco System with forced infusion was used, we did not observe above-mentioned problem. During one operation, there was need for wound enlargement and the lens was implanted through 2.0 mm incision. Even though in our study, no sign of intraoperative or postoperative inflammation could be seen, which indicates that this technique seems to be safe.

It is widely known that the smaller the incision the lower SIA [14, 15]. In comparison with coaxial surgery biaxial surgery shows significantly less SIA [14, 15]. B-MICS procedure allows sub-1.8 mm incisions, which effectively decrease the induction or changes in corneal SIA during cataract surgery in comparison with C-MICS [14]. Alio et al. [16] suspect that “2.0 mm is the limit around which no optical changes are induced by cataract surgery in the human cornea.” In present study SIA was equal 0.29 ± 0.16 D and was very similar to the Jimenez et al. result: 0.31 ± 0.22 D, *p* > 0.05 [8]. There was a statistically significant difference between our and Sonnleithner et al. [5] and Toygar et al. [7] results, where SIA was equal 0.45 ± 0.29 and 0.45 ± 0.28, respectively (*p* < 0.05). Probable reasons behind these differences might be the wound stretching during IOL implantation [5] and the incision size, which was equal 1.8 mm in Toygar et al. [7] study. Cavallini et al. [17] demonstrated that more posterior wound retraction is observed in 1.8-mm incisions compared to that in 1.4-mm incision (53 vs. 47%). It seems that the limit around which no optical changes are induced by cataract surgery is lower and might be sub-1.8 mm.

In our study, CDVA before surgery improved significantly from 0.58 ± 0.57 to −0.05 ± 0.06 in third month and was stable during 12-month follow-up. These results were significantly better compared with other study results on Incise IOL (*p* < 0.05) [5–7]. Jiménez et al. [6] reported improvement of mean DCVA from 0.57 ± 0.15 to 0.16 ± 0.13 3 months postoperatively, Sonnleithner et al. [5] from 0.4 ± 0.27 to 0.05 ± 0.07 4 weeks after surgery, Toygar et al. [7] from 0.52 ± 0.42 to 0.01 ± 0.02 logMAR 6 months postoperatively.

In present study, 1 year after the surgery mild PCO was observed especially in the peripheral part of the capsule, but the center was clear in all eyes. In consequences, no one patient needed YAG-capsulotomy 1 year after surgery. It is reasonable to conclude that lack of significant PCO was connected with design of the IOL: continuous 360 square-edge profile, which prevents PCO formation. According to study results of Nanavaty et al. [18] who evaluated 3 different IOLs: 2 aspheric microincision hydrophilic IOLs (Acri.Smart 36A and Akreos MI-60) and conventional single piece hydrophobic acrylic spheric IOL (AcrySof SN60AT; Alcon Laboratories), there is a trend in hydrophilic IOLs toward progression over the 2-year follow-up. At 2 years, the mean PCO score was lower than 11% for the conventional AcrySof IOL and 23% for the Akreos MI-60 IOL. Longer observation time is necessary to assess frequency of significant PCO formation.

Incise IOL has high level of predictability and is comparable with other IOLs [19]. Toygar et al. [7] reported 6 month after surgery 94% of eyes were within 1.0 D from calculated refraction in comparison with our 90% 1 year after surgery.

Other advantage of microincision is low number of higher-order aberration (HOA). It is commonly known that HOA might be caused of decreased visual acuity and contrast sensitivity [5]. In study, results by Sonnleithner et al. [5] outcome in terms of Incise IOL HOA were very satisfying and comparable to other aspheric IOLs (Akreos MI60, Tecnis ZCB00, CT Asphina). In our study, the results of contrast sensitivity for distance were within normal limits of healthy people in the same range of age indicated very good performance of this type of IOL. Our results agree with those in other study [6] on Incise IOL. Additionally, the follow-up period was 6 months longer in our study.

The patients were highly satisfied with the quality of performed procedures and implanted lenses due to the fact that they received mostly very good, uncorrected visual acuity for distance. None of patients complained about glare. In our experiences with other IOL implanted using B-MICS procedure [20, 21], glare effect was also not observed.

In summary, the results of presented study suggest that B-MICS with Incise lens implantation is a procedure which provides for the patient very good visual function as well as high patient’s satisfaction. So, we would recommend the B-MICS and this type of IOLs for the cataract surgeons and patients. It seems reasonable to expect that Incise lens might be a good platform for introduction for multifocal IOL.
